# Does the Prevalence of Dyslipidemias Differ between Newfoundland and the Rest of Canada? Findings from the Electronic Medical Records of the Canadian Primary Care Sentinel Surveillance Network

**DOI:** 10.3389/fcvm.2015.00001

**Published:** 2015-02-02

**Authors:** Shabnam Asghari, Erfan Aref-Eshghi, Oliver Hurley, Marshall Godwin, Pauline Duke, Tyler Williamson, Masoud Mahdavian

**Affiliations:** ^1^Memorial University of Newfoundland (MUN), St. John’s, NF, Canada; ^2^University of Calgary, Calgary, AB, Canada; ^3^University of Ottawa, Ottawa, ON, Canada

**Keywords:** dyslipidemia, Canada, Newfoundland, lipid profile, prevalence, Canadian Primary Care Sentinel Surveillance Network

## Abstract

**Introduction:** Newfoundland and Labrador (NL) has the highest prevalence of cardiovascular disease (CVD) in Canada. Dyslipidemia is a risk factor for CVD. This study compares the prevalence of dyslipidemia in the NL population with the rest of Canada.

**Methods:** A cross-sectional study, using data from the Canadian Primary Care Sentinel Surveillance Network (CPCSSN), was undertaken. The study population included adults, excluding pregnant women, aged 20 years and older. Canadian guidelines were used for classifying dyslipidemia. Univariate and multivariate analyses were conducted to compare the lipid levels and prevalence of dyslipidemia between NL and the rest of Canada.

**Results:** About 128,825 individuals (NL: 7,772; rest of Canada: 121,053) were identified with a mean age of 59 years (55% females). Mean levels of total cholesterol (4.96 vs. 4.93, *p* = 0.03), low-density lipoprotein (LDL) (3.00 vs. 2.90 mmol/L, *p* < 0.0001), triglyceride (1.47 vs. 1.41 mmol/L, *p* < 0.0001), and high-density lipoprotein (HDL) (1.29 vs. 1.39 mmol/L, *p* < 0.0001) were significantly different in NL compared to the rest of Canada. Dyslipidemias of LDL (29 vs. 25% *p* < 0.0001), HDL (38 vs. 27%, *p* < 0.0001), and triglyceride (29 vs. 26%, *p* < 0.0001) were significantly more common in NL. After adjustment for confounding variables, NL inhabitants were more likely to have dyslipidemia of total cholesterol (OR: 1.16, 95% CI: 1.10–1.23, *p* < 0.0001), HDL (OR: 1.52, 95% CI: 1.44–1.60, *p* < 0.0001), LDL (OR: 1.38, 95% CI: 1.30–1.46, *p* < 0.0001), and ratio (OR: 1.53, 95% CI: 1.42–1.60, *p* < 0.0001).

**Conclusion:** The NL population has a significantly higher rate of dyslipidemia compared to the rest of Canada, and the mean levels of all lipid components are worse in NL. Distinct cultural and genetic features of the NL population may explain this, accounting for a higher rate of CVD in NL.

## Introduction

Cardiovascular diseases (CVDs) are the leading cause of death worldwide, resulting in more than 17.3 million deaths in 2008 (1). This number is expected to reach 23.3 million in 2030 (2). Geographic variations in cardiovascular mortality have frequently been reported in previous studies. According to the 2008 report by WHO, the age standardized CVD mortality rates in the developed world (Australia, Japan, France, and United States) has been 100–200/100,000 population in contrast to higher rates in the rest of the world (3). The 2014 update on the burden of CVD reports significant regional differences in the age standardized cardiovascular mortality rates across the European continent, including lower rates of 150–180 deaths/100.000 in western European countries (Netherlands, Norway, France, and Switzerland), higher rates among southern countries of Europe such as Greece (246.7/100.000) and Cyprus (219.2/100.000), and rates of above 500/100.000 in eastern European countries (Russia, Romania, and Ukraine) (4).

In Canada, CVD is the main cause of death at 32% and, after musculoskeletal diseases, has the highest economic burden of disease (5). Compared to other provinces, Newfoundland and Labrador (NL) has the highest level of CVD morbidity and mortality in adults. Between 1995 and 1998 in NL, age standardized CVD rates per 100,000 populations ranged from 320.6/100,000 to 196.9/100,000 (6). During the same period, NL had the highest life expectancy lost (LEL) due to CVD – more than 5.3 years – in all of Canada (7). According to a Statistics Canada report in 2007, NL had the highest age standardized mortality rate for major CVDs among all Canadian provinces: 218.5/100,000 population in NL compared to a nationwide average mortality rate of 151.9/100,000 (8). The NL CVD mortality rates are more similar to those of southern European countries as well as low and middle income countries, which have a higher mortality rates compared to the developed world (3, 4).

Although regional variations in CVD and CVD morality have been recognized, the explanation for these differences is still unclear. Some studies suggest that these variations could be related to the variations in cardiovascular risk factors including smoking, obesity, hypertension, and dyslipidemia (9, 10). Dyslipidemia, defined as abnormal blood lipid levels, includes elevated total cholesterol, low-density lipoprotein (LDL), and triglyceride, and decreased high-density lipoprotein (HDL) is one of the most important and well-known risk factors of CVDs (11). The risk for CVD conferred by dyslipidemia varies according to the type of condition and presence or absence of other risk factors. It has been reported to be one of the most important determinants of myocardial infarctions among adults of young age (12). Dyslipidemia is a multifactorial trait wherein environmental and behavioral factors, as well as genetic predisposition, play a role (13).

Although Canada, in general, has a diversity of cultures and ethnicities, the present NL population almost entirely originates from 20,000 migrants from south-west England and the south of Ireland in the mid-1700s (14). This founding population experienced a low level of in-migration over the centuries, which resulted in NL being one of the few remaining isolated Caucasian populations worldwide (14). This isolation has shaped a unique and homogenous culture and genetic background, both of which may contribute to the prevalence of complex traits such as dyslipidemia and, consequently, CVD (14). It is also notable that there is anecdotal evidence from family physicians in NL, who worked in different provinces that a different pattern of lipid profiles and a higher prevalence of dyslipidemia exists in NL compared to other Canadian provinces.

Dyslipidemia in NL has been previously documented in several reports. School-aged children in the 1980s had higher total cholesterol levels compared to age, sex, and racially matched American children (15). A 1990s’ study on the rural Newfoundland population found that 61% of the subjects had hypercholesterolemia recorded in their medical records (16). A recent study on a sample of 4,424 primary health-care patients in NL reported that 42% of the patients had high cholesterol, 36% high LDL, 25% low HDL, and 25% abnormal triglyceride; however, the results were not compared with other populations (17). The lack of comparison groups, lack of biochemical measurement, and cohorts, which were not fully representative of the NL population were limitations of these studies. The purpose of the current study is to compare the prevalence of dyslipidemias in residents of NL and compare these with the rest of Canada.

## Materials and Methods

### Source of data

The Canadian Primary Care Sentinel Surveillance Network (CPCSSN) is a pan-Canadian network that extracts data from the electronic medical records (EMRs) of family physicians. At the time of this study, the CPCSSN database included data from close to 600 primary care clinicians in rural and urban settings across 10 provinces of Canada. CPCSSN is Canada’s first library of digital information based on point-of-care data from primary care practices. Data from these EMRs are extracted quarterly and uploaded in a de-identified format to both regional and central (pan-Canadian) databases. The databases are used for chronic disease surveillance in primary care and are also used as a tool for conducting primary care research (18). At the time of this study, the pan-Canadian CPCSSN database included 844,592 individuals over 20 years of age, corresponding to 3% of the Canadian population; the NL component included 46,588 individuals, representing 11% of the NL population (18).

### Study population

All adults over 20 years of age (excluding pregnant women) from the CPCSSN database who had a lipid profile in the CPCSSN database between January 1, 2010 and December 31, 2012 were included in the study. To identify pregnant women, the text and/or ICD code records for every event related to pregnancy were queried (19).

### Variables

#### Lipid variables

Canadian guidelines for the diagnosis and management of dyslipidemia (20), the nationwide protocol for all practitioners in Canada, suggests a lipid screen for all men over 40 years of age, all women of over 50 years, all postmenopausal women, all individuals with diabetes, hypertension, obesity, first degree relative with history of CVD under the age of 60, as well as current smokers. The routine screening test requires the measurement of all lipid components.

In this study, the most recent lipid profiles (total cholesterol, HDL, LDL, and triglyceride) for each individual were recorded. The ratio of total cholesterol to HDL was calculated by dividing total cholesterol by HDL. Dyslipidemia was defined using the Canadian guidelines for the diagnosis and treatment of dyslipidemia (Table 1) (20). Individuals with at least one abnormal component were classified as those with any dyslipidemia.

**Table 1:** **Healthy levels of serum lipids for Canadian adults (20–23)**.

Total cholesterol	<5.2 mmol/L (20–79 years)
Triglyceride	<1.7 mmol/L
Low-density lipoprotein-cholesterol (LDL)	<3.4 mmol/L
High-density lipoprotein-cholesterol (HDL)	>1.0 mmol/L men; >1.3 mmol/L women
Ratio of total cholesterol to HDL	<5.0

#### Geographic variables

In Canada, a national six-digit postal code is used to identify a geographic location. Each postal code is unique and represents a location in the real world. The first digit is specific to each province, whereas the second one classifies the region as rural or urban. Accordingly, we used the first digit of the postal codes to separate those patients living in NL from those in the rest of Canada, and the second digit to classify individuals as rural or urban residents (24). The rural/urban residence was included in the multivariate analysis as previous studies suggest that the CVD risk factors could differ between the rural and urban inhabitants (25).

#### Covariates

To account for confounding factors, CVD risk factors as well as other variables with effect on lipid levels were extracted from the EMR (20). The demographic variables included age and gender. Obesity was defined as BMI ≥30; whereas those with BMI lower than 30, but higher than 25 were classified as overweight. The smoking status was extracted from the most recent record by the family physician at the time of the lipid test, and individuals were classified as non-smokers, past smokers, and current smokers. CPCSSN algorithms for chronic conditions were used to ascertain both diabetes and hypertension (26). These algorithms have high sensitivity and specificity to detect diabetes and hypertension (26). Diagnostic text and ICD code records related to these conditions in EMRs were used for other chronic conditions, including dyslipidemia and CVD (27, 28). Medication use was identified using the text record of the medication name and/or anatomical therapeutic chemical (ATC) codes. Usage of lipid modifying agents (HMG-CoA reductase inhibitors, fibrates, bile acid sequestrants, nicotinic acid, and other agents) was stratified into three categories: current users (any record of lipid-lowering medication use before the date of a blood test and continuing until up to the time of the blood test); previous users (record of drug use between 2 years and 3 months before the date of the blood test); and non-users (no drug use for the last 2 years before the date of a blood test). The classification of medication use here has been previously used in other studies involving the study of lipid profile databases (29). Medications with unintended effects on lipid levels (30) including thiazides, loop diuretics, beta blockers, alpha blockers, ACE inhibitors, calcium channel blockers, estrogen, progesterone, hormone replacement therapy, and corticosteroids were extracted from EMRs.

### Statistical analysis

Characteristics of the study population, as well as the mean and confidence intervals of the individual lipid components, were summarized using descriptive statistics. Classical tests of hypothesis including Student’s *t*-test and the chi-squared test were conducted to test for the association between variables. Logistic regression modeling was used to examine the association between dyslipidemia and living in NL while controlling for age, sex, rurality, and other potential influential factors. For variables with more than 5% of missing information, i.e., smoking (~70% missing) and BMI (~50% missing), a code for missing values was considered wherever model based analyses were performed. In the model, age was classified into three groups and each group was compared against the oldest one (Age > 65); smoking variables were compared with non-smokers; overweight and obese patients were compared with normal and underweight individuals; non-medication users and previous medication users were compared with current users as baseline. A subgroup analysis was also performed to investigate how the patients of EMR primary care in NL differ from the patients of EMR primary care in the rest of Atlantic Canada. A *p*-value <0.05 was considered statistically significant. All analyses were performed using STATA/SE 11.2 (Stata Corp., College Station, TX, USA). Geographical variation in the prevalence of dyslipidemia was also presented using ArcMap 10.0 (Build 4000).

### Ethics

The study protocol was approved for ethics by Health Research Ethics Authority (HREA) of Newfoundland and Labrador.

## Results

### Population description

Among the 430,169 individuals recorded in the CPCSSN database from January 1, 2010 to December 31, 2012, 128,825 individuals (~30%) had completed blood testing and met the study criteria. The mean age was 59 years, 55% were women, and 77% were living in an urban area. The majority of the population (78%) was not taking any lipid-lowering medications, whereas 8 and 14% were categorized as previous users and current users, respectively. Among the drug users, HMG-CoA reductases (Statins) were the most commonly used form of lipid-lowering drugs (94%), while other drug consumption was rare (data not shown). Table 2 presents general characteristics of the study population in NL and the rest of Canada.

**Table 2:** **Characteristics of study population in Newfoundland and Labrador and the rest of Canada**.

Characteristics	Newfoundland and Labrador (%) (*n* = 7,772)	Rest of Canada (%) (*n* = 121,053)	*p*-value
Age [(mean (95% CI)]	58.3 (58.0–58.6)	59.2 (59.1–59.3)	<0.0001
Body mass index [(mean (95% CI)]	30.3 (29.8–30.9)	28.1 (28.0–28.1)	<0.0001
Sex (female)	55.8	50.1	0.70
Residence (rural)	25.1	22.6	<0.0001
Smoking (current)	22.4	13.5	<0.0001
Smoking (past)	22.1	42.8	<0.0001
Hypertension	33.5	34.0	0.39
Diabetes	16.2	15.3	0.04
History of dyslipidemia	39.9	20.7	<0.0001
Cardiovascular disease	44.9	35.4	<0.0001
Lipid-lowering medication (current users)	20.5	13.6	<0.0001
Lipid-lowering medication (previous users)	8.0	8.0	0.95

*CI, confidence interval*.

### Comparison of lipid levels and dyslipidemia in NL with the rest of Canada

**Figure 1:**
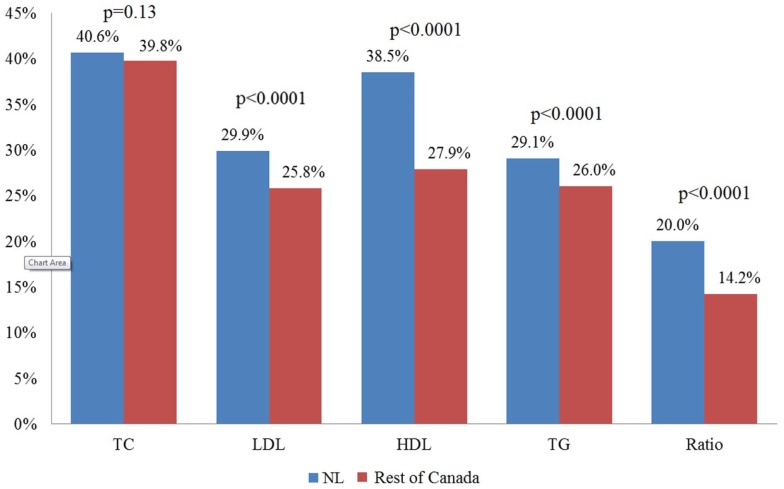
**Percentage of adults with unhealthy levels of lipids in patients of primary care in Newfoundland and Labrador and the rest of Canada. NL, Newfoundland and Labrador**.

Mean levels for all lipid components in NL and the rest of Canada are presented in Table 3. There are significant differences between NL and the rest of Canada for all of the lipid components. Approximately 72% of the NL population have at least one abnormal lipid component (any dyslipidemia), whereas this figure is 64% in the rest of Canada (*p* < 0.0001). Figure 1 illustrates the prevalence of dyslipidemia in NL and the rest of Canada. Prevalence of abnormal LDL (29 vs. 25%), HDL (38 vs. 27%), triglyceride (29 vs. 26%), and ratio (20 vs. 14%) were significantly higher in NL (*p* < 0.0001). The greatest difference was observed for HDL dyslipidemia with an 11% difference (Figures 1 and 3).

**Table 3:** **Mean levels of lipid components in Newfoundland and Labrador and the rest of Canada [mean (95% confidence interval)]**.

	Newfoundland and Labrador	Rest of Canada	*p*-value (*t*-test)
Total cholesterol	4.96 (4.94–4.98)	4.93 (4.93–4.94)	0.034
Low-density lipoprotein	3.00 (2.98–3.02)	2.90 (2.89–2.91)	<0.0001
High-density lipoprotein	1.29 (1.28–1.30)	1.39 (1.39–1.40)	<0.0001
Triglyceride	1.47 (1.45–1.49)	1.41 (1.40–1.42)	<0.0001
Ratio	4.06 (4.04–4.09)	3.76 (3.75–3.77)	<0.0001

### Comparison of dyslipidemia in NL with rest of Canada controlling for covariates

Table 4 shows the results of the multivariate logistic regression model for dyslipidemia. After adjusting for the effect of covariates, NL inhabitants were more likely to have dyslipidemia of total cholesterol (OR: 1.16, 95% CI: 1.10–1.23, *p* < 0.0001), HDL (OR: 1.52, 95% CI: 1.44–1.60, *p* < 0.0001), LDL (OR: 1.38, 95% CI: 1.30–1.46, *p* < 0.0001), and total cholesterol/HDL ratio (OR: 1.53, 95% CI: 1.42–1.60, *p* < 0.0001).

**Table 4:** **Multivariable logistic regression models assessing lipid abnormalities between Newfoundland and Labrador compared to the rest of Canada [odds ratio (95% confidence interval)]**.

	Total cholesterol	LDL	HDL	Triglyceride	Total cholesterol/HDL ratio
Newfoundland and Labrador	1.16 (1.10,1.23)	1.38 (1.30,1.46)	1.52 (1.44,1.60)	1.03 (0.98,1.09)^NS^	1.53 (1.44,1.63)
Sex (females)	1.70 (1.65,1.75)	1.00 (0.97,1.03)^NS^	1.90 (1.85,1.96)	0.59 (0.57,0.61)	0.35 (0.34,0.37)
Age (40–65)	0.92 (0.89,0.95)	1.06 (1.03,1.10)	1.39 (1.34,1.44)	1.20 (1.16,1.24)	1.84 (1.77,1.91)
Age (<40)	0.38 (0.36,0.40)	0.54 (0.51,0.57)	2.10 (1.99,2.23)	1.39 (1.31,1.48)	1.66 (1.55,1.77)
Past smokers	1.03 (0.98,1.09)^NS^	1.03 (0.97,1.09)^NS^	1.02 (0.96,1.09)^NS^	1.02 (0.96,1.08)^NS^	1.00 (0.92,1.08)^NS^
Current smokers	1.03 (0.95,1.11)^NS^	1.17 (1.08,1.27)	1.45 (1.34,1.58)	1.59 (1.47,1.72)	1.84 (1.67,2.02)
Overweight	1.14 (1.08,1.20)	1.49 (1.40,1.58)	1.82 (1.70,1.95)	2.23 (2.07,2.4)	1.86 (1.72,2.01)
Obese	0.86 (0.82,0.91)	1.34 (1.26,1.43)	2.99 (2.79,3.20)	4.13 (3.85,4.44)	2.96 (2.74,3.19)
Rural residence	0.86 (0.83,0.89)	0.91 (0.88,0.94)	1.09 (1.05,1.13)	1.29 (1.25,1.34)	1.22 (1.17,1.26)
Diabetes	0.32 (0.31,0.34)	0.39 (0.37,0.42)	1.68 (1.61,1.75)	2.47 (2.37,2.57)	1.07 (1.02,1.12)^c^
Hypertension	0.86 (0.83,0.89)	0.88 (0.84,0.91)	1.00 (0.96,1.04)^NS^	1.46 (1.40,1.51)	1.14 (1.09,1.19)
Cardiovascular disease	0.91 (0.88,0.95)	0.93 (0.89,0.97)	1.13 (1.09,1.18)	1.08 (1.04,1.13)	1.05 (1.01,1.10)^c^
Lipid-lowering (previous user)	1.88 (1.75,2.01)	2.08 (1.91,2.27)	0.96 (0.90,1.02)^NS^	0.96 (0.91,1.02)	1.59 (1.47,1.73)
Lipid-lowering (non-user)	5.35 (5.08,5.63)	4.78 (4.48,5.10)	0.80 (0.76,0.83)	0.51 (0.49,0.53)	2.13 (2.00,2.26)
Drugs with unintended lipid effects	1.03 (1.00,1.07)^NS^	1.01 (0.97,1.05)^NS^	1.07 (1.03,1.11)	1.01 (0.98,1.05)^NS^	0.94 (0.90,0.98)^c^
Low-density lipoprotein	–	–	0.72 (0.71,0.73)	–	–
Triglyceride	2.14 (2.10,2.18)	1.59 (1.56,1.63)	2.67 (2.62,2.73)	–	–
High-density lipoprotein	–	1.34 (1.29,1.40)	–	–	–
Total cholesterol	–	–	–	1.93 (1.90,1.96)	–

*All the presented odds ratios are significant at *p* < 0.0001 except for ^c^*p* < 0.05, ^NS^not significant (*p* > 0.05)*.

High-density lipoprotein dyslipidemia was significantly more common in adults <40 years old (OR: 2.10, 95%CI: 1.99–2.23, *p* < 0.0001) compared to other age groups. The greatest effect on the dyslipidemias of all components was observed by lipid-lowering medications (except for HDL and triglyceride) followed by age and body mass index. Drugs with unintended lipid effects had no significant influence on lipid levels. Further analysis on the lipid levels was performed using linear regression modeling and findings with similar trends were observed (data not shown).

### Comparison of dyslipidemia between NL and Atlantic Canada

Our data identified 25,409 individuals from Atlantic Canada, New Brunswick, Nova Scotia, Prince Edward, and NL. The NL population had significantly higher levels of total cholesterol (4.96 vs. 4.83, *p* < 0.0001), LDL (3.00 vs. 2.88, *p* < 0.0001), and total cholesterol/HDL ratio (4.06 vs. 3.99, *p* = 0.04) compared to the rest of Atlantic Canada. No significant difference was observed between NL and the other Atlantic Canadian provinces for triglyceride and HDL levels.

**Figure 2:**
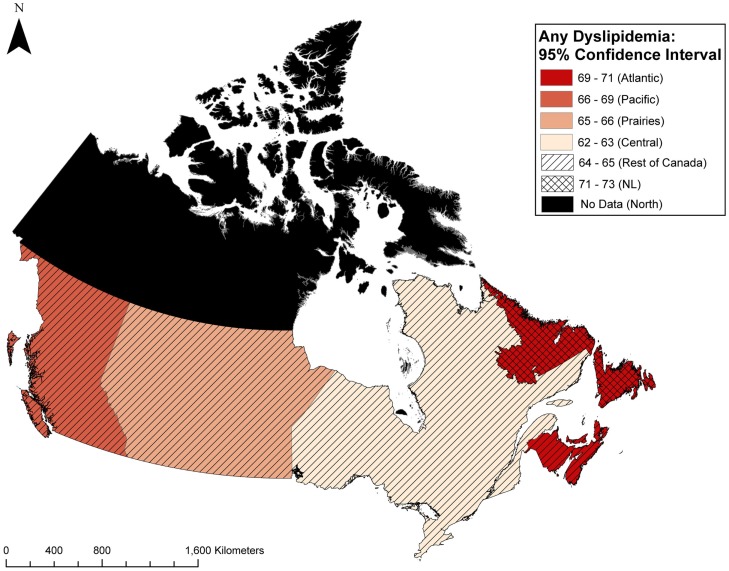
**Geographic distribution of those with any dyslipidemia in primary care patients in Canada**.

**Figure 3:**
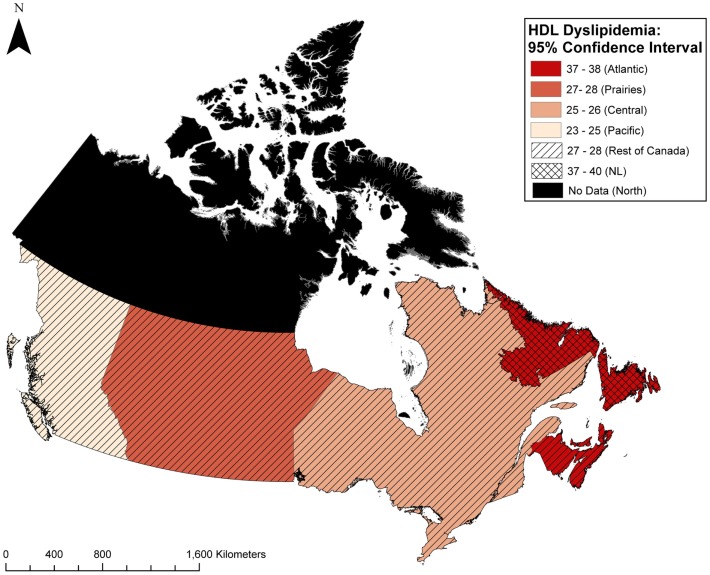
**Geographic distribution of HDL dyslipidemia in primary care patients in Canada**.

Compared to the rest of Atlantic Canada, the NL population had a higher prevalence of total cholesterol dyslipidemia (40 vs. 37%, *p* < 0.0001), HDL dyslipidemia (38 vs. 37%, *p* = 0.04), LDL dyslipidemia (30 vs. 27%, *p* < 0.0001), and non-significant trends toward higher triglyceride dyslipidemia, and ratio dyslipidemia. The results did not change after adjustment for covariates using multivariate logistic regression (data not shown). Distribution of those with any dyslipidemia as well as HDL dyslipidemia in these patients in NL and the four Canadian regions including Atlantic Canada, Central Canada, Prairies, and Pacific Canada are illustrated in Figures 2 and 3, respectively.

## Discussion

Our study included a large population of individuals who visited family physicians in NL and across Canada. To our knowledge, this is the first study comparing biochemical measurements of lipid profiles between two large populations from NL and the rest of Canada. The prevalence of dyslipidemia in NL is significantly higher compared to the rest of Canada with the greatest difference found for HDL dyslipidemia. This might suggest that the actual differences in dyslipidemia are much higher than what was observed in our study. HDL levels are not generally influenced by the common therapeutic agents administered for the management of dyslipidemia (21). This is also consistent with the finding from multivariate models in our study where a weak association is reported between lipid-lowering medications and HDL. Moreover, the adults in our study who were younger than 40 years of age were more likely to have lower HDL level compared to the adults of other age groups. Although this might be related to the possibility that the younger individuals in our study are from a high risk group, further investigation of the associated factors of lower HDL level among this age group could be an area of concentration for future studies.

This study has some limitations. It is a secondary analysis of EMR data from primary care practices in Canada. EMR data are collected for clinical practice and the extent to which this data could be accurate for research could be questioned. Furthermore, we used a cross-sectional study design, which meant to provide a snapshot of dyslipidemia in Canada. The casual/temporal relationship between lipid abnormalities and their risk factors should be interpreted with caution. The negative association between dyslipidemia and morbidities including CVD, diabetes, and hypertension should be interpreted in the line of a therapeutic regime suggested by the CVD prevention guidelines for patients with those conditions (20).

Electronic medical records are becoming more commonly used in medical practice; however, the quality of EMR data may not be optimal. A systematic review on use of EMR for health outcome research suggests that the validity of EMR data differs from country to country and from health condition to health condition (31). A recent study by Tu et al. assessed the completeness of primary care EMR data in Ontario, Canada. The study shows good capture of information and low level of missing information within primary care EMRs compared with administrative data (32). Another study using data from CPCSSN shows high validity and reliability for eight common chronic conditions in primary care CPCSSN data. The study suggests that CPCSSN data are a suitable source for health service research (33).

Statistics Canada surveys could be a complement to the EMR data that we used in the study, as the best measure for examining the discrepancies in lipid profiles between different communities and geographic regions. Especially, if it is argued that our results only correspond to a portion of the population who had a lipid profile conducted by their family physician; generally speaking, a population with higher morbidity.

This study, however, is the first of its kind to compare a large population from NL and the rest of Canada (11 and 3% of the overall population, respectively), and the findings show strong similarities with lipid levels of Canadians as reported from the Canadian Health Measure Surveys (22, 23, 34, 35), in serum levels of total cholesterol (4.94 vs. 4.93), LDL (2.91 vs. 2.90), HDL (1.39 vs. 1.41), triglyceride (1.42 vs. 1.35), and ratio (3.79 vs. 3.77) as well as the prevalence of dyslipidemia (22) of total cholesterol (both 40%), LDL (both 26%), HDL (28 vs. 25%), and ratio (14 vs. 16%), respectively. Findings from our previous study in NL, using secondary data from NL laboratories on a sample of 94,000 adults 20 years and older who had lipid profile tests between 2009 and 2010, also showed similarities with the present study (36). Both of these reports support differences in lipid levels and the higher prevalence of dyslipidemia in NL, particularly for HDL dyslipidemia.

### Implications for clinical practice

The fundamental research idea came from experienced clinicians in several Canadian provinces who suspected an abnormality in Newfoundlanders’ lipid profiles. Our results are in agreement with their speculations. The observed differences in the prevalence of dyslipidemia in our study will have clinical benefits in designing guidelines for the treatment and prevention of dyslipidemia and CVD, as well as in identifying the contributing factors in a distinct population such as NL, where the current therapeutic and prevention guidelines might not be as effective and applicable. This is particularly of importance for HDL-C, which is known to be a key determinant of CVD risks, which even persists among patients with low levels of LDL-C (37).

The findings in our study are also consistent with previous studies suggesting regional differences in the prevalence of dyslipidemia in different regions of Europe (38) and among Hispanic and non-Hispanic whites populations in USA (39). These studies suggested a more aggressive management of the risk factors in the afflicted areas. Such a strategy could also be considered by the authorities in NL province of Canada.

## Conclusion

Analyses of the CPSSN data indicate a significantly higher prevalence of adverse lipid components in NL compared to the rest of Canada, including a higher prevalence of low HDL in NL.

It is difficult to determine exactly what the lower HDL levels in younger adults and unhealthy levels of lipids in adults generally mean for CVD prevalence in the future as the individuals age; however, it may be an indication that the high rate of CVD in NL is not likely to change any time soon. The results of this study provide more evidence of our theory that a different pattern of serum lipids is present in NL, which has a homogenous population, and suggests that more research into lipid profiles is required, especially with regard to the effects of HDL on cardiovascular health in that population. Our findings will be beneficial in designing guidelines for the treatment and prevention of dyslipidemia and CVD, as well as in identifying the contributing factors specific to NL population.

## Conflict of Interest Statement

The authors declare that the research was conducted in the absence of any commercial or financial relationships that could be construed as a potential conflict of interest.
